# The effect of *Bongardia Chrysogonum* on prostate tissue in a rat model of STZ-induced diabetes

**DOI:** 10.1186/s40064-016-2973-z

**Published:** 2016-08-11

**Authors:** Recep Dokuyucu, Kerem Han Gozukara, Oguzhan Ozcan, Nebihat Kaplan Sefil, Ahmet Nacar, Ahmet Dokuyucu, Mehmet Inci

**Affiliations:** 1 Department of Medical Physiology, School of Medicine, Mustafa Kemal University, 31100 Antakya, Hatay Turkey; 2 Department of Urology, School of Medicine, Mustafa Kemal University, Hatay, Turkey; 3 Department of Biochemistry, School of Medicine, Mustafa Kemal University, Hatay, Turkey; 4 Department of Histology, School of Medicine, Mustafa Kemal University, Hatay, Turkey; 5 Department of Histology, School of Medicine, Hacettepe University, Ankara, Turkey; 6 Department of Information Technology, Lumina-The University of South-East Europe, Bucharest, Romania

**Keywords:** Diabetes mellitus, Prostate, *Bongardia chrysogonum*, Testosterone, Oxidative stress, Inflammation

## Abstract

**Background:**

*Bongardia chrysogonum* is widely used in Turkey for treating urinary tract infections and prostate hypertrophy, and it also has potent hypoglycemic effects and aids glucose homeostasis. Because of the inflammatory conditions in diabetes mellitus (DM), the prostate tissue of men with diabetes is particularly susceptible to developing hypoplasia, and DM produces characteristic pathological changes in prostate tissue. Here, we examined the effects of *B. chrysogonum* on the prostate tissue of rats with streptozotocin (STZ)-induced diabetes.

**Results:**

The glucose levels were statistically significantly higher in the diabetic rats than in healthy controls (P < 0.001). Further, they were significantly lower in the healthy and diabetic rats administered *B. chrysogonum* than in the untreated diabetic rats (P < 0.001 and 0.05, respectively). The total cholesterol levels were significantly lower in the healthy rats administered *B. chrysogonum* than the healthy controls (P < 0.05) and diabetic rats (P < 0.01). They were also significantly lower in the diabetic rats administered *B. chrysogonum* than those that were left untreated (P < 0.05). The testosterone levels were significantly lower in the untreated diabetic rats than in the controls (untreated ones and those administered *B. chrysogonum*) and diabetic rats administered the herb (P < 0.001, 0.05 and 0.01, respectively). The oxidative stress index was significantly higher in the untreated diabetic rats than the healthy controls (P < 0.05). It was also significantly lower in the healthy and diabetic groups treated with *B. chrysogonum* than the untreated diabetic rats (P < 0.05). Histological examination showed no changes in the prostate tissue of the non-diabetic rats. In the diabetic group, the glandular lumens were filled with cellular debris and leucocytic infiltrate, and the glandular epithelium was degenerated and thickened. In the diabetic group treated with *B. chrysogonum*, the epithelium was better preserved and less debris was seen in the glandular lumen.

**Conclusion:**

To our knowledge, this is the first study to histologically prove the effects of *B. chrysogonum* on prostate tissue in diabetes. Our findings may be useful in developing *B. chrysogonum* into a therapeutic agent against diabetes and benign prostate hyperplasia.

## Background

Benign prostatic hyperplasia (BPH) is characterized by narrowing of the urethral opening as a result of the growth of prostate tissue around the urethra (COMMİTTEE 2003). Histological BPH is observed in 8 % of men between 31 and 40 years of age, the incidence increases markedly with age, and reaches about 90 % in the 9th decade of life (Rosen et al. 2003, 2005), and this condition greatly hampers quality of life. Although most literature implicates non-controllable factors like age, hormonal status, and genetics in BPH, newer publications link it to metabolic factors such as high blood glucose and blood lipid levels, and the field is rapidly advancing (Parsons 2007). Thus, new targets for prevention and treatment of BPH may be revealed in the near future.

As with BPH, the incidence of diabetes mellitus (DM) increases with age, and developed communities need to allocate a large share of healthcare expenses toward these diseases. Between 3.5 and 4.2 million men in the United States have both BPH and DM. When treating either of these diseases, however, the possible presence of other pathologies must be kept in mind, as the population most commonly affected by them is the elderly (Klein et al. 2005).

*Bongardiachrysogonum* is a tuberous plant used in the treatment of certain diseases in south-east Anatolia. Its use particularly in the treatment of urinary tract infections, prostate hypertrophy, hypercholesterolemia, epilepsy, and cancer has increased its national demand, and it is marketed throughout Turkey (Arslan et al. 2005). *B. chrysogonum* also reportedly has potent hypoglycemic effects and is useful for glucose homeostasis, in addition to multiple anti-inflammatory effects exerted via saponins (Arslan et al. 2005). Saponins are fat- and water-soluble glycosides that combine with glycan or aglycan components of *B. chrysogonum*. They inhibit the emulsification of fat molecules and bile acids via reducing their surface tension and detergent effects in the gastrointestinal tract. Another well-defined anti-hypercholesterolemic mechanism of saponins is the ability to change the quantity and dimension of cholesterol micelles, which reduces their absorption by intestinal mucosa cells (Rahman et al. 2000; Rao and Kendall 1986; Sidhu and Oakenfull 1986; Whitehead et al. 1981), with consequent effects on metabolism.

In the present study, we examined the effects of *B. chrysogonum* on prostate tissue in a rat model with streptozotocin (STZ)-induced diabetes in terms of the histological and biochemical aspects.

## Methods

The ethics committee of Mustafa Kemal University approved the animal use protocol for this study, which is in compliance with the Declaration of Helsinki (ID: 40595970/126). Male Wistar albino rats were used (250–300 g) in this study. They were housed in a 12 h light and dark environment at room temperature. During the experiment, the rats were provided normal mouse pellets and water. Throughout the experiment, the animal conditions were maintained according to international ethical guidelines for laboratory animals.

### Animal protocol

Forty adult male Wistar albino rats were divided into four groups [control (C), diabetes group (D), *B.chrysogonum* group (B), and diabetes + *B. chrysogonum* group (DB)] of 10 rats each. During the test period, the rats in all groups were given normal mouse pellets and water. At the beginning of the experiment, the blood sugar levels in all animals were measured using a glucometer. The rats in groups D and DB were administered 60 mg/kg STZ intravenously (prepared by dissolving STZ in citrate buffer). After 72 h, the urine of rats from groups D and DB was examined for glucose levels using strip analyzers. Rats with blood glucose concentrations ≥300 mg/dL (and for the urine samples, the color of the strip changed from yellow to green) were considered diabetic (Gulturk et al. 2010).

### Preparation of *B. chrysogonum* infusion

*Bongardia chrysogonum* obtained from a local herbalist was crushed, and an infusion was prepared by passing 100 mL of boiled water through 3 g of this material. A fresh infusion was prepared each week before the test phase (Arslan et al. 2005).

### Administration of *B. chrysogonum*

The rats in groups B and DB were administered 0.2 mL of the infusion obtained as mentioned above by oral gavage once every day for 5 weeks (Arslan et al. 2005). The rats in groups C and D were similarly administered 0.2 mL of water. At the end of the 5-week experiment, all rats were anesthetized using ketamine (90 mg/kg) and xylazine (3 mg/kg).

### Hematological evaluation

Cardiac blood samples were collected, and the serum levels of glucose, total cholesterol (TC), high-density lipoprotein (HDL), low-density lipoprotein (LDL), and triglycerides (TG) were measured using spectrophotometry.

### Measurement of total antioxidant status

Serum total antioxidant status (TAS) levels were measured using the new automated method developed by Erel (2004). This assay has excellent precision (<3 %). In this method, the most powerful hydroxyl radicals are generated first. Reagent 1 containing ferrous ion solution is mixed with reagent 2, which includes hydrogen peroxide, n the assay. The brown-colored dianisidine radical cation is a strong radical produced by hydroxyl radicals. Non-potent radical reactions initiated by the hydroxyl radicals produced because of antioxidative effect of working examples were also measured. The results are expressed in mmol Trolox equivalents/L.

### Measurement of total oxidant status

Serum total oxidative status (TOS) levels were also measured using the method of Erel (2005). In this method, oxidants present in the samples oxidize the ferrous ion-o-dianisidine complex to ferric ions. The oxidation reaction is enhanced by abundant glycerol molecules in the reaction medium. The ferric ions form a colored complex with xylenol orange in an acidic medium. The color intensity, which can be measured spectrophotometrically, is related to the total amount of oxidant molecules in the sample. The assay was calibrated using hydrogen peroxide. The results are expressed in terms of micromoles of hydrogen peroxide equivalents per liter (μmol H_2_O_2_ equivalents/L).

### Oxidative stress index

The percentage of the TOS to TAS ratio was used as the oxidative stress index (OSI) value [OSI = TOS (μmol H_2_O_2_ equivalents/L)/TAS (mmol Trolox equivalents/L)] (Dokuyucu et al. 2014).

### Histopathological assessment

Renal and prostate samples were fixed in 10 % neutral buffered formalin for light microscopic examination. After the tissues were dehydrated in a graded alcohol series and embedded in paraffin, 5-μm-thick transverse sections were prepared from the tissue blocks and stained with hematoxylin–eosin and reticulin for histologic evaluation. Sections were examined for characteristic changes and photographed using an Olympus DP20 camera attached to an Olympus CX41 photomicroscope (American Diabetes Association 1998; McVary 2006a, b).

### Statistical analysis

Statistical analysis was performed using GraphPad software, version 5.0 (GraphPad Inc., La Jolla, CA, USA). Normally distributed continuous variables were tested using one-way ANOVA and the post hoc Student’s *t* test. P values less than 0.05 were considered statistically significant.

## Results

### Glucose, TC, TG, HDL, LDL, and testosterone levels

**Fig. 1:**
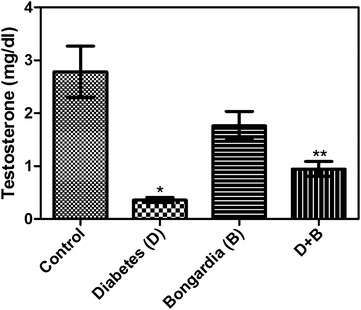
Comparison of the testosterone levels in groups

The glucose levels were significantly higher in group D than in group C (P < 0.001). Further, they were significantly lower in groups B and DB than in group D (P < 0.001 and 0.05, respectively) (Table 1). No significant differences were found in the TC, TG, LDL, or HDL levels between groups D and C. TC levels were significantly lower in group B than groups C and D (P < 0.05 and 0.01, respectively). They were also significantly lower in group DB than group D (P < 0.05). The LDL levels were significantly lower in group DB than in group D (P < 0.05) (Table 1). Lastly, the testosterone levels were significantly lower in group D than groups C, B, and DB (P < 0.001, 0.05 and 0.01, respectively) (Table 1; Fig. 1).

**Table 1:** Laboratory data and levels of oxidative status in groups (mean ± SE)

	Control	Diabetes (D)	Bongardia (B)	D + B
Glucose (mg/dL)	234.1 ± 16.72	584.4 ± 71.6^a^	195.4 ± 39.36^b^	307.6 ± 51.8^c^
T. cholesterol (mg/dL)	61.91 ± 7.63	61.52 ± 3.79	42.95 ± 3.04^c,d^	45.77 ± 5.00^e^
Triglycerids (mg/dL)	59.97 ± 11.00	56.42 ± 6.01	49.11 ± 5.68	52.68 ± 7.04
HDL (mg/dL)	16.01 ± 1.84	15.74 ± 1.46	10.72 ± 1.66	13.55 ± 1.15
LDL (mg/dL)	30.94 ± 4.31	34.43 ± 3.48	25.68 ± 4.11	22.88 ± 2.45^e^
TAS (mmol/L)	0.50 ± 0.03	0.66 ± 0.03	0.79 ± 0.08^d^	0.61 ± 0.05
TOS (mmol/L)	7.63 ± 1.11	13.79 ± 1.21^d^	9.44 ± 1.02	7.97 ± 1.85^e^
OSI (=TOS/TAS)	10.47 ± 1.18	19.15 ± 1.97^d^	12.00 ± 1.76^e^	12.94 ± 0.90^e^
Testosterone (mg/dL)	2.78 ± 0.48	0.36 ± 0.05^a^	1.76 ± 0.27^e^	0.94 ± 0.14^f,c^

*HDL* high-density lipoprotein, *LDL* low-density lipoprotein, *TAS* total antioxidant status, *TOS* total oxidative status, *OSI* oxidative stress index

^a^p < 0.001

^b^p < 0.001

^c^p <  0.01

^d^p < 0.05

^e^p < 0.05 vs. diabetes

^f^p < 0.01 vs. control

### TAS, TOS, and OSI results

**Fig. 2:**
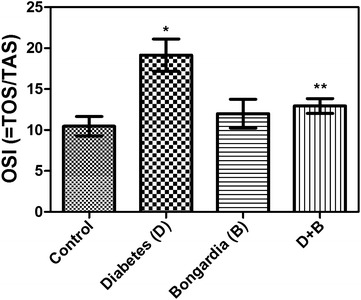
Comparison of the oxidative stress index in groups

The TAS levels were significantly higher in group B than group C (P < 0.05), while the TOS levels were significantly higher in group D than groups C and DB (P < 0.05). The OSI levels were significantly higher in group D than group C (P < 0.05). They were also significantly lower in groups B and DB than group D (P < 0.05) (Table 1; Fig. 2).

### Histopathological staining

**Fig. 3:**
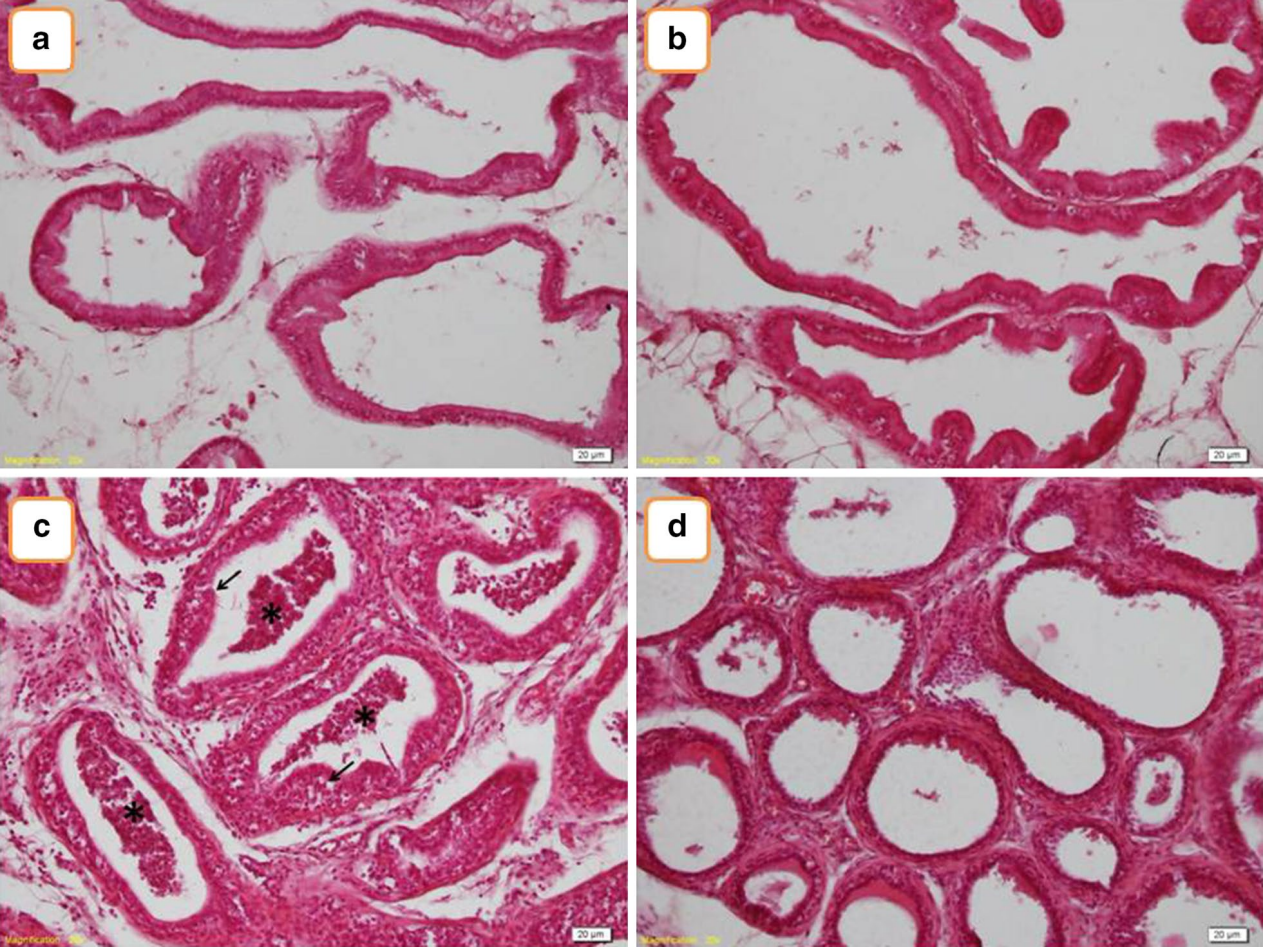
Comparison of the histopathologic parameters in groups. **a** Control group; **b** diabet group; **c** bongardia group; **d** diabet+bongardia group

Microscopic examination showed that the prostate tissue was normal in groups C (Fig. 3a) and B (Fig. 3b). In group D, the glandular lumens were filled with cellular debris and leucocytic infiltrate (Fig. 3c). The glandular epithelium was also degenerated or thickened in some regions. Administration of *B. chrysogonum* improved the general tissue structure and the epithelium was better preserved and less debris was seen in the glandular lumen in group DB than in group D (Table 2; Fig. 3d).

**Table 2:** The histopathology scores of prostate tissues (mean ± SE)

	Control	Diabetes (D)	Bongardia (B)	D + B
Leucocytic infiltrate	0.20 ± 0.13	2.40 ± 0.5^a^	0.20 ± 0.12^c^	1.30 ± 0.15^b,d^
Epithelial degeneration	0.10 ± 0.10	2.50 ± 0.16^a^	0.20 ± 0.13^c^	1.12 ± 0.13^b,d^

^a^p < 0.0001

^b^p < 0.01, vs. control

^c^p < 0.0001

^d^p < 0.01, vs. diabetes

## Discussion

Inflammation is a generalized histopathologic finding of BPH (Fibbi et al. 2010; Schauer and Rowley 2011) and is associated with a higher risk of lower urinary tract symptoms (LUTS) (Kramer and Marberger 2006) and acute urinary retention in aging men (Nickel et al. 2008). Recent studies have shown an increased risk of BPH in patients proven to have prostate inflammation on biopsy examination (Crawford et al. 2006). In prostate tissue developing hyperplasia, inflammation can induce proliferation events and nuclear deterioration through cytokines and oxidative stress (Naber and Weidner 2000). The aggressive release of oxygen radicals from macrophages and neutrophils during acute or chronic inflammation may lead to compensatory cellular proliferation, which further exacerbates oxidative stress, and in such conditions, hyperplastic proliferation may occur in the prostate tissue (Vital et al. 2015). Tanik et al. (2014) used inflammatory parameters like the neutrophil-to-lymphocyte ratio to predict BPH progression and LUTS severity and found a positive correlation between the neutrophil count and prostate hyperplasia.

High-fat diets (HFD) have also been implicated in oxidative stress and inflammation in the prostate gland (Shankar et al. 2012) as they increase the levels of acute-phase inflammatory markers (Kriketos et al. 2004) via cytokines released from neutrophils and macrophages in adipose tissue (Shankar et al. 2015). Using a HFD-induced metabolic syndrome animal model with hyperglycemia, dyslipidemia, and hypogonadism, Vignozzi et al. (2012) linked metabolic syndrome to prostate inflammation, and found that it was correctible with testosterone replacement. Like HFD, DM has also been linked to chronic inflammatory conditions, and it has been implicated in increasing cytokine levels, mostly in the adipose tissue (Gibb and Strachan 2014). Further, DM-related androgen deficiency has been observed in a third of the population with type 2 diabetes, although the underlying mechanism remains unclear (Cheung et al. 2015; Morton 2005). The prostate volume has been found to be especially high in male patients diagnosed with type 2 diabetes (Kasturi et al. 2006), and in the NHANES-3 cohort study, Rohrman et al. observed a higher incidence of BPH in men with diabetes than in those without diabetes (American Diabetes Association 1998). Our results confirmed the effects of DM on prostate tissue shown in previous literature. A continuous supply of androgen is crucial for the growth and normal functioning of the prostate gland, and when this supply stops or decreases, prostate disease may develop (Yadav and Heemers 2012).

Rahman et al. (2000) conducted a detailed chemical examination of *B. chrysogonum* and examined the effects of saponins extracted from these tubers. Saponins are fat- and water-soluble glycosides with glycan or aglycan structures. In the intestine, saponins form micelles with bile acids, vitamins, fat acids, and diglycerides. Several studies have shown that because of a reduction in surface tension and detergent effects in the gastrointestinal tract, saponins reduce the emulsification of fat molecules and bile acids (Rao and Kendall 1986; Sidhu and Oakenfull 1986; Whitehead et al. 1981). Further, Bingham et al. (1978) showed that although saponins decreased the serum cholesterol and liver lipid concentrations, the liver cholesterol and serum HDL levels remain unchanged. The findings of our study agree with those of previous studies, as we clearly observed the anti-cholesterolemic and anti-lipidemic effects of *B. chrysogonum* in the blood and liver, respectively. Further, the testosterone levels in group D of the present study were significantly lower than those in group DB. These findings indicate that the hypogonadal status induced by DM was ameliorated by administration of *B. chrysogonum*. Additionally, correction of the deficiency in testosterone levels, which exacerbates prostate inflammation and tissue remodeling, may prevent or at least decelerate the course of BPH.

Arslan et al. (2005) and Assaf et al. (2013) studied the effects (Fig. 3) of *B. chrysogonum* on various tissues under certain conditions. However, to our knowledge, ours is the first study to histologically examine its effects on prostate tissues in DM.

Histologically at the cellular level, BPH is characterized by basal cell hyperplasia, increased stromal mass, enhanced extracellular matrix deposition, reduced elastic tissue, increased infiltration of leucocytes around ducts, and acinar hypertrophy (Bostwick et al. 1992).

The accumulation of inflammation throughout the prostatic tissue aggravates the process of prostatic hyperplasia by enhancing the production of prostatic growth factors. Furthermore, the altered secretion of luminal cells and clogging of the ducts with debris and corpora amylacea may lead to prostatic calcification, which is a significant modification of the hyperplastic prostate. Our findings showed that the conditions of the prostate tissue were better in rats with diabetes who were treated with *B. chrysogonum* than in those that were not treated with this herb: as mentioned in the “Results” section, the epithelium was better preserved and less debris was seen in the glandular lumen in group DB than in group D. Further, the TOS levels were significantly higher in group D than group DB, while the OSI levels were significantly lower.

Chronic conditions such as DM and hypercholesterolemia require lifelong treatment, and patient compliance may be better with alternative herbal treatment options in the case of such conditions. As indicated by our findings, the well-known plant *B. chrysogonum* seems to have positive effects on glucose and lipid metabolism. Further, it seems to prevent the pathological changes that occur in the prostate tissue in DM. Thus, this herb may be a useful treatment option for patients with DM and BPH. Further molecular studies are needed to confirm the effects of *B. chrysogonum* on the prostate tissue and to develop this herb into an effective therapeutic agent against DM and BPH.

## Abbreviations

DM: diabetes mellitus
BPH: benign prostate hyperplasia
TOS: total oxidative status
TAS: total antioxidant status
OSI: oxidative stress index
TC: total cholesterol
TG: triglycerides
LDL: low-density lipoprotein
HDL: high-density lipoprotein
HFD: high-fat diet
STZ: streptozotocin
LUTS: lower urinary tract symptoms
